# A Voice from Texas

**Published:** 1884-10

**Authors:** 


*Publishers.*

### A VOICE FROM TEXAS.

The following extract taken from a private letter from Dr. F. E. Daniel, editor of the Texas *Courier-Record*, shows in what esteem *The Journal* is held in the Lone Star State:

Your September number of ATLANTA MEDICAL AND SURGICAL JOURNAL to hand. Accept our congratulations on the excellence of matter and mechanical execution, typography, etc. It is very handsome indeed. Your ads make a better appearance than any we know. But what has more particularly attracted my attention and admiration is the "artotype," of the grand old Irish Georgian. We all love him, and his genial countenance sheds sunshine even in a picture. Your enterprise is commendable, not only in this but in your "Army Surgeons;" shall watch that with increasing interest, inasmuch as I was a Confederate surgeon, served in Georgia many months.

### ITEMS.

The JOURNAL had a pleasant visit, a few days since, from Dr. S. H. Gray, a prominent physician of Barnesville, Ga.

Dr. R. A. Lewis, of Richmond, Virginia, has taken charge of the department of "Abstracts and Gleanings" of the *Southern Clinic.*

In the last *Medical News*, of September 27, Dr. Hilsman, of Albany, Ga., reports a case of death from chloroform inhalation.

Dr. J. S. Todd has moved into his elegant new residence at 74 Marietta street. He now has the handsomest offices in the city.

Dr. Jas. F. Alexander has been elected a member of the Board of Health of this city, made vacant by the death of Dr. W. G. Drake.

Dr. H. F. Campbell, of Augusta, President of the American Medical Association, is on a trip North in the interest of the Association.

The *Century*, for October, has been received. It presents an unusually attractive table of contents. It is the best literary magazine we know.

The prospects for a large class at the Atlanta Medical College during the coming session, which begins October 9th, are very flattering indeed.

Dr. John H. Logan will deliver the introductory address at the opening of the Atlanta Medical College, instead of Dr. Taliaferro, as stated in our last.

Dr. J. McF. Gaston has been elected to the chair of Surgery in tha Southern Medical College, made vacant by the resignation of Prof. Jno. Thad. Johnson.

Drs. A. Jacobi and N. S. Davis were appointed at Copenhagen as the American representatives on the International Collective Investigation of Disease Committee.

Dr. W. G. Drake, of this city, died on 11th of September, of Bright's disease, after an illness of several months. He was a member of the Board of Health of the city.

Dr. J. Berrien Lindsey has been elected Secretary and executive officer of the Tennessee State Board of Health, in place of Dr. C. C. Fite, resigned.

Dr. H. V. M. Miller, who has been sojourning in the mountains of Western North Carolina for the past three months, has returned, looking very much improved by his trip.

The Jefferson Medical College, of Philadelphia, has elected Dr. J. W. Mallett, of the University of Virginia, to the chair of Chemistry in that school, to succeed the late Prof. Robert E. Rogers.

The published report of an English benevolent society says: "Notwithstanding the large amount paid for medicine and medical attendance, very few deaths occurred during the year."—*Ex.*

Dr. J. C. Avary, of this city, and Miss Myrta Lockett were married at the home of the bride, near Petersburg, Va., on September 17th. The JOURNAL extends its congratulations to the happy pair.

Dr. Thad. A. Reamy, of Cincinnati, the most prominent gynecologist in the West, was in Atlanta a few days ago and called to see us. We regret we were out at the time and failed to see him.

In London a man fell in a drunken fit and broke his neck. The jury found out that his grandfather had died of a broken neck, and brought in as their verdict, "Died by the hereditary visitation of God."—*Ex.*

We begin in this number of the JOURNAL the publication of the proceedings of the Macon Medical Society and of the Atlanta Medical and Surgical Union. The more important papers and the discussions will appear regularly every month hereafter.

The Macon Society is probably the oldest medical society in the State, having been in successful operation for almost twenty years. Its first president was the venerable Dr. D. W. Hammond. Its present officers are Dr. C. H. Hall, President· Dr. H. J. Williams, Secretary, and Dr. H. McHatton, Reporter. The Atlanta Medical and Surgical Union has been organized for a few months only, but gives every promise of becoming a prosperous organization. Its officers are Dr. W. S. Elkin, President, and Dr. J. F. Lancaster, Secretary.

Dr. Samuel J. Davis, of Roanoke, Ala., writes us of a case of hydrophobia which occurred in his practice recently. The case died on the fifty-sixth day after inoculation. For seventy-two hours previous to death the patient did not sleep one moment.

"The Care and Feeding of Infants," is the title of a handsomely printed pamphlet issued by Messrs. Doliber, Goodale & Co, Boston. While it is an advertisement of Mellins' food for infants, it nevertheless contains a great deal of useful information. A copy of the pamphlet will be sent free to any one ordering it from the publishers.

The following testimonial of a patent medicine speaks for itself: "Dear Sir—Two months ago my wife could scarcely speak. She has taken two bottles of your 'Life Renewer,' and now she can't speak at all. Please send two more bottles. I wouldn't be without it."

We give an unusual space in this issue to Pasteur's address on infectious diseases and vaccination for rabies, delivered at the International Medical Congress at Copenhagen. We feel sure that the address will be read with interest by every one, coming as it does from the foremost man in the medical world to day.

We give space in this issue of the JOURNAL to a communication addressed to the Medical Association of Georgia by Dr. Henry Gaither, of Oxford. While we agree with the Doctor that something ought to be done to increase the membership of the Association, and thereby increase its usefulness, we do not believe the measures advocated by him will accomplish the desired result.

In the September issue of THE JOURNAL we mentioned the fact that Dr. J. McF. Gaston had organized a surgical infirmary in this city. We are pleased to know that the Doctor is meeting with encouragement in his new enterprise. Dr. Gaston was for ten years superintendent of a similar institution in Brazil. The infirmary is located at 107 Marietta street.

To stimulate fraternity, the *Philadelphia Medical World* suggests that the Northern delegates to the next meeting of the American Medical Association rendezvous at Louisville or St. Louis and charter a steamer for the trip both ways, and that the passengers use the steamer as a hotel while at New Orleans. While for some this mode of travel would consume too much time, those who can adopt this plan can be assured of an enjoyable trip.—*Med. & Sur. Reporter.*

The *Cincinnati Lancet* gives the record of the new member of the Board of Health of Cincinnati. It quotes verbatim from the Police Court docket. Since 1878 he has been before the court six times, three times for assault and battery, and three times for disorderly conduct. Twice he was convicted and four times discharged. Is such the training required for a reputable, efficient member of Cincinnati's health board?

A correspondent of the *Medical News* says that an operation of enterotomy was recently performed upon a Pittsburgh lady of wealth. The surgeon removed four inches of the small intestine, uniting the divided ends by sutures. She recovered. He charged $1,000 for the operation and twelve days' attendance. As payment was refused he brought suit. The jury gave a verdict of $330, which was thus arrived at: Each put down the amount he would allow. They then divided the aggregate sum by twelve, and thus reached the verdict.

*The Weekly Medical Review* announces that, after the first of January, it will be enlarged, and that a special department of obstetrics and gynecology will be added, under the editorial management of Dr. G. J. Engelmann, of St. Louis. It will then be known as "*The Weekly Medical Review and Journal of Obstetrics and Diseases of Women.*" The special department will be paged separately, so that it can be bound by itself. We congratulate the *Review* on having secured Dr. Engelmann's services, and trust that the success of its proposed plan will be complete.

In 1871 there was 300,000 deaths from cholera in Russia; in 1873 there were 16,000 deaths in Poland; in 1872-73 there were 140,000 deaths in Hungary; in 1872-73 there were nearly 27,000 deaths in Prussia; in 1865-67 there were 143,000 deaths in Italy. In Paris the mortality from cholera has been as follows: In 1832, 18,654

deaths; in 1849, 19,184; in 1853-54, 8,096; in 1865-66, 12,082; in 1873, 885. In England, in 1849, the deaths from cholera were 70,-000. In 1817 the army of the Marquis of Hastings in India lost 9,000 men in twelve days from Asiatic cholera.

DIDN'T CATCH HIS MEANING.—The New York *Sun* says that a Chinaman came into the ladies' cabin of a Brooklyn ferry boat the other day and took a seat beside an Irish market woman. He seemed to want to make himself agreeable, and remarked: "Belly cold." The woman looked at him with an air of contempt, and replied: "If you'd put your shirt in your pants your belly wouldn't be cold, you haythen blackguard."

The American Public Health Association begins its eleventh annual meeting at St. Louis, October 14th, and continues four days. The subjects designated by the committee for special consideration are: Hygiene of the Habitations of the Poor; Hygiene of Occupations; School Hygiene; Adulteration of Food; Water Pollution; Disposal of Sewage by Chemical Action; The Observable Effects upon Public Health of Official Supervision, and the Work of Municipal and State Boards of Health. It is believed that the meeting will be one of the largest ever held.

Dr. Julius Wise proposes to publish, during the coming year, a large volume under the title "Encyclopedia of Medical Wit, Humor, and Curiosities of Medicine." In this undertaking he respectfully solicits the kindly aid of the profession. Witticisms and anecdotes of a humorous or curious nature are solicited. Physicians druggists, dentists and others supplying original contributions will receive due credit in the work. The author is especially anxious to avail himself of every source, and would highly appreciate all information concerning publications likely to be useful for reference. All letters, contributions, clippings, books and other matter should be addressed to Julius Wise, M. D., 806 Olive street, St. Louis, Mo.

A friend of THE JOURNAL gives us the following: A patient came to me a few days ago who had been treated by an irregular but legal practitioner, in this city, for stricture of the urethra. The patient stated that the doctor had told him that the redness around the meatus was a sure indication of the size of the stricture and that he would remove it "by the roots" by using something on the bougies, which were used daily. The patient was required to furnish the doctor with a specimen of the urine every day that he might look out for the "roots."

